# Overexpression of *Kif1A* in the Developing *Drosophila* Heart Causes Valvar and Contractility Defects: Implications for Human Congenital Heart Disease

**DOI:** 10.3390/jcdd7020022

**Published:** 2020-06-02

**Authors:** Takeshi Akasaka, Karen Ocorr, Lizhu Lin, Georg Vogler, Rolf Bodmer, Paul Grossfeld

**Affiliations:** 1 Development, Aging & Regeneration Program, Sanford Burnham Prebys Medical Discovery Institute, La Jolla, CA 92037, USA; akasaka-cba@umin.ac.jp (T.A.); gvogler@sbpdiscovery.org (G.V.); rolf@sbpdiscovery.org (R.B.); 2 Cardiology and Catheterization Laboratories, Shonan Fujisawa Tokushukai Hospital, Fujisawa City 251-0041, Japan; 3 Division of Cardiology, Department of Pediatrics, UCSD School of Medicine, San Diego, CA 92093, USA; lilin@health.ucsd.edu

**Keywords:** *Kif1A*, cardiac development, congenital heart disease, aortic valve, *Drosophila*, myoblasts

## Abstract

Left-sided congenital heart defects (CHDs) are among the most common forms of congenital heart disease, but a disease-causing gene has only been identified in a minority of cases. Here, we identified a candidate gene for CHDs, *KIF1A*, that was associated with a chromosomal balanced translocation t(2;8)(q37;p11) in a patient with left-sided heart and aortic valve defects. The breakpoint was in the 5′ untranslated region of the *KIF1A* gene at 2q37, which suggested that the break affected the levels of *Kif1A* gene expression. Transgenic fly lines overexpressing *Kif1A* specifically in the heart muscle (or all muscles) caused diminished cardiac contractility, myofibrillar disorganization, and heart valve defects, whereas cardiac knockdown had no effect on heart structure or function. Overexpression of *Kif1A* also caused increased collagen IV deposition in the fibrous network that normally surrounds the fly heart. *Kif1A* overexpression in C2C12 myoblasts resulted in specific displacement of the F-actin fibers, probably through a direct interaction with G-actin. These results point to a Kif1A-mediated disruption of F-actin organization as a potential mechanism for the pathogenesis in at least some human CHDs.

## 1. Introduction

Congenital heart defects are the most common birth defect, occurring in about 0.7% of all newborn infants [1]. Left-sided heart defects comprise one of the most common forms of congenital heart disease and have a strong genetic component [2]. One of the most severe heart defects, hypoplastic left heart syndrome (HLHS), occurs in 2–4% of all infants born with congenital heart disease, and there are ~1000–2000 infants diagnosed each in year in the United States with HLHS [3]. Despite significant strides in palliative surgical approaches for HLHS, in which the right ventricle is converted to function as the systemic ventricle, HLHS still accounts for 20–25% of mortality in all infants born with congenital heart disease. The long-term prognosis for survivors remains guarded, and it is likely that many patients will ultimately require cardiac transplantation.

Although there are multiple lines of evidence supporting a genetic etiology for left-sided heart defects, the underlying molecular and cellular mechanisms are poorly understood. The most commonly held theory is that, in HLHS, left ventricular hypoplasia results secondarily due to hemodynamic perturbations, specifically decreased anterograde blood flow into the developing left ventricle, secondary to obstruction of the mitral and/or aortic valves. In support of this hypothesis, ligation of the left atrium in the chick embryo decreased left ventricular inflow and causes hypoplasia of the left ventricle [4]. Alternatively, in some cases, HLHS may be due to a primary defect in left ventricular development. In this case, HLHS may represent a severe neonatal form of cardiomyopathy, suggesting an underlying defect in cardiac myocyte development. Consistent with this model, histopathologic analysis of left ventricular myocardial tissue from explanted HLHS infant hearts revealed fibrosis and myocyte disarray [5], as observed in some forms of cardiomyopathy, although it is possible that these changes arise secondarily to impaired hemodynamics. Interestingly, mutations in the cardiac transcription factor NKX2.5 have been observed in patients with HLHS [6] and in other patients with hypertrophic cardiomyopathy (HCM) [7], suggesting that HLHS and HCM could represent different forms of cardiomyopathy along a common disease spectrum. In this case, it is possible that multiple developmental pathways are affected that cause primary valve and myocardial defects through a “multi-hit” mechanism (either through a single or multiple affected genes) and that additional genetic and/or epigenetic factors can influence the severity of the phenotype.

In this study, we identified a patient with left-sided congenital heart defects who carried a balanced chromosomal translocation t(2;8)(q37;p11). We localized the chromosome 2 breakpoint to the 5′ putative regulatory region of the *KIF1A* gene. The KIF protein superfamily comprises microtubule-associated molecular motors that are critical for intracellular transport [8,9,10]. Mutations in other KIF family members (KIF3A and KIF7) have been associated with sinus inversus and heterotaxy [11], suggesting that KIF proteins are essential for normal heart development and that impaired expression of other members of the KIF family might also cause congenital heart defects. In adults, *Kif1A* has been shown to be dysregulated following myocardial infarction [12] and a *Kif1A* motor domain mutation has been associated with spastic paraplegia [13]. We hypothesized that dysregulation of *KIF1A* expression contributed to the left-sided heart defects in our patient. In this study, we demonstrate that overexpression (OE) of *Kif1A* causes cardiac morphological and functional defects in the fly heart that are consistent with defects observed in F-actin organization and maturation of cultured mammalian myoblasts. *Kif1A* OE also altered bristle arrangement and ommatidium viability in the fly, suggesting an effect on cell polarity. Taken together, these data demonstrate that dysregulation of *Kif1A* expression may impair normal heart development via disruption of F-actin organization, suggesting a potentially novel mechanism for some of the most common forms of human congenital heart disease.

## 2. Materials and Methods

### 2.1. Patient Consent

Consent to obtain blood and to perform genetic studies was obtained through a University of California – San Diego Institutional Review Board-approved protocol.

### 2.2. Analysis of Human Aortic Valve Tissue

For histological analyses, human aortic valve was dissected out into single leaflet and fixed in 37% formalin, washed with Phosphate Buffered Saline (PBS), dehydrated with ethanol, embedded in paraffin, and cut into 10-µm-thick sections using a microtome. Sections were stained with hematoxylin-eosin, Masson’s trichrome stain, and von korssa stain according to standard protocols. An age- and sex-matched control sample, obtained through the San Diego County Coroner’s Office, was used from a patient who died from noncardiac causes.

### 2.3. Fly Stocks

We used “driver” fly lines expressing the following alleles: twist-Gal4;24B-Gal4, GMH5, GMR-Gal4, pannier-Gal4, Mef2–Gal4, and Hand-Gal4. Overexpression of the mouse *Kif1A* transgene was performed using the UAS-Gal4 system, where “driver”-Gal4 lines were crossed to transgenic lines containing an upstream activating (UAS) sequence and either mouse *Kif1A* or interfering RNA against unc-104 (UAS unc104 RNAi). The yw laboratory strain was used as a reference in this study. To generate the *Kif1A* transgenic flies, the mouse *Kif1A* was subcloned into the pUAST *Drosophila* transformant vector (details below) and then injected according to standard procedures. Three individual transformant lines were examined in this study (line 3, line 6, and line 8b). Unc-104 RNAi line (transformant ID #23455) was obtained from the Vienna *Drosophila* RNAi center (Vienna, Austria).

### 2.4. Fly Heart Staining and Image-Based Heart Performance Assay

Flies were anaesthetized with FlyNap© (Carolina Biological Supply Co, Burlington, NC, USA) followed by dissection in artificial hemolymph as previously described [14], and cardiac function was assayed using high speed digital imaging (100–150 fps, 9300 EM-CCD camera, Hamamatsu Corp., Bridgewater, NJ, USA) followed by movie analysis with the Semiautomatic Optical Heartbeat Analysis (SOHA; www.sohasoftware.com) method [15,16].

### 2.5. Quantitative RT-PCR

Total RNA was extracted from whole flies (N = 6), or isolated thoraxes (N = 10), abdomens (N = 10), heads (N = 20), intestines (N = 20), and hearts (N = 30) (the same number of male and female flies per group) using TRIzol (Invitrogen, Carlsbad, CA, USA) and purified with RNeasy kit (QIAGEN, Germantown, MD, USA) for adult fly or Mini RNA isolation kit (Zymo Research, Irvine, CA, USA) for heart RNA. After treatment with DNase I, first strand cDNA was transcribed by SuperScript III (Invitrogen, Carlsbad, CA, USA) by oligo(dT) primer, followed by second strand synthesis. Quantitative PCR was carried out using LightCycler FastStart DNA Master PLUS SYBR Green I kit (Sigma-Aldrich Corp., St. Louis, MO, USA). The following primer sets were used:

rp49: 5′- GACGCTTCAAGGGACAGTATCTG -3′ 5′- AAACGCGGTTCTGCATGAG -3′ 

unc-104: 5′- GCGCTGCTATAACTCAG-3′ 5′- GTTTGTTCCGTAACGTGTTG -3′

### 2.6. Cloning of Full-Length and Truncated Mouse Kif1A

The mouse *Kif1A* cDNA (m*Kif1A*/pYX-Asc) was purchased from Clontech (Mountain View, CA, USA). Site-directed mutagenesis to convert the terminal codon into a BamHI site was performed by using the QuikChange Site-Directed Mutagenesis kit (Stratagene, La Jolla, CA, USA) using the following oligonucleotides (the base pair substitution is underlined):

5’-CTGCGCAGATGCGGGTC*GGATCC*GAAAGCCTTCCGGACTTC-3’ and

5’- GAAGTCCGGAAGGCTTTC*GGATCC*GACCCGCATCTGCGCAG -3’.

Full-length *Kif1A* was subcloned into the EcoRI-BamH1 site of the pcDNA3.1 myc-His(-) vector to add the myc tag and then subcloned into the XbaI-Not1 site of the pUAST vector. Three truncated proteins containing the motor domain (MD), the forkhead associated domain (FAD), and the Pleckstrin homology domain PH were obtained using full-length mouse *Kif1A* as a template. The fragments were amplified by PCR using the following primer sets:

MD: 5’-CGAGAAATGGCCGGGGCCTCTG-3’ and 5’GTCGACCAGCCAGCAAGGCTTCCCTCT-3’

FAD: 5’- CTCGAGAGATGGGTGTGGCCATGAG-3’ and

5’-GTCGACCACGGTGGTCCACCACAGCA-3’

PH: 5’- CTCGAGGCATGGGGACTTTCCTTCTC-3’ and

5’-GTCGACTCTGCGCAGATCTCCTTCGA-3’

The PCR fragments were ligated into a pCR2.1-TOPO cloning vector (Invitrogen, Carlsbad, CA, USA) and sequenced to ensure no errors were introduced during the PCR. All the fragments of full-length and truncated Kif1A were then subcloned into the XhoI-NotI site of pcDNA3.1/myc-His(-)A expression vector (Invitrogen, Waltham, MA, USA) for cell transfection experiments.

### 2.7. Cell Culture and Transfection

C2C12 cells were cultured in Dulbecco’s modified Eagle’s medium (D-MEM) supplemented with 15% (*v*/*v*) heat-inactivated fetal bovine serum in a humidified 5% CO2 incubator at 37 °C. For transient transfections, C2C12 cells were grown on collagen I coated cover slips in 6-well plate and transfected with 1 µg of myc-tagged *KifA* expression plasmids using 4 µL of FuGene 6 transfection reagent (Roche) according to the standard protocol provided by the manufacture. Thirty-six hours after transfection, cells were immunostained (see below). C2C12 cells were differentiated into myocytes at 24 h after transfection in D-MEM supplied with 2% (*v*/*v*) heat-inactivated horse serum and Insulin-Transferrin-Selenium liquid media supplement (Sigma). After 36 h in differentiation medium, the cells were immunostained (see below).

### 2.8. Immunostaining

Cells were fixed with 4% Paraformaldehyde for 15 min and permeabilized with 0.5% Triton X100 in PBS for 5 min at room temperature. Cells were blocked with 3% Bovine Serum Albumin in PBS followed by primary antibody incubation at room temperature for 2 h. Mouse anti-myc at 1:100 (Santa Cruz Biotechnology, Dallas, TX, USA), mouse anti-tublin at 1:500 (Sigma-Aldrich, St. Louis, MO, USA), and Mouse anti-GFP (Green Fluorescent Protein) at 1:250 (Invitrogen, Carlsbad, CA, USA) were used for primary antibodies. Cells were then incubated with FITC (Fluorescein isothiocyanate) -conjugated donkey anti-mouse IgG (1:200; Jackson ImmunoResearch Laboratory, West Grove, PA, USA) with Alexa Fluor 594-conjugated phalloidin (1:100; Invitrogen, Carlsbad, CA, USA) was at room temperature for 2 h. Cells were mounted on glass slides with Vectashield (with DAPI), and the images were taken using a Fluoview 1000 Olympus Laser Point Scanning Confocal Microscope. Myocyte cell sizes and nuclei number were quantified from confocal images using Image J Trainable Weka Segmentation plugin.

Semi-intact fly heart preparations were prepared for immunohistochemistry by first incubating in 10 mM EGTA (ethylene glycol-bis(β-aminoethyl ether)-N,N,N′,N′-tetraacetic acid) in artificial hemolymph for 5 min to arrest the heart in the diastolic phase. Then, hearts were fixed with 4% paraformaldehyde and processed as previously described [17]. Images were taken using a Mouse anti-pericardin (collagen IV) antibody (Developmental Studies Hybridoma Bank, Univ. of Iowa) at 1:100 for primary antibody, and cells were then incubated with 488 Alexa Fluor-conjugated goat anti-mouse IgG (1:200; Jackson ImmunoResearch Laboratory, West Grove, PA, USA) and with Alexa Fluor 594-conjugated phalloidin (1:100; Invitrogen, Carlsbad, CA, USA) to stain F-actin. Images were taken with a Zeiss ApoTome microscope (Carl Zeiss, White Plains, NY, USA). The pericardin area was quantified from Z stack projections using Image J and the Trainable Weka Segmentation plugin.

### 2.9. Immunoprecipitation and Western Blot

HEK293T cells were co-transfected with 3 µg of full-length or truncated myc-tagged *Kif1A* and 3 µg of GFP-tagged actin or GFP expression plasmids in 10-cm culture dish using 24 ugl of FuGene6 (Sigma-Aldrich Corp., St. Louis, MO, USA).

Thirty-six hours after transfection, cells were lysed on ice for 30 min with a lysis buffer (20 mM Tris PH7.6, 150 mM NaCl, 1% NP40, and 1 mM EDTA, Ethylenediaminetetraacetic acid) supplemented with CompleteMini proteinase inhibitor (Sigma-Aldrich Corp., St. Louis, MO, USA). Lysates were centrifuged to remove the insoluble fraction and supernatants were pre-cleaned with Protein G sepharose (Sigma-Aldrich Corp., St. Louis, MO, USA). The supernatants were then incubated with 1 µg of rabbit anti-myc antibody (Santa Cruz Biotechnology Inc, Dallas, TX, USA) for 2 h at 4 °C followed by incubation with Protein G sepharose for one hour at 4 °C. After 6 washes with lysis buffer, bound proteins were eluted at 70 °C for 5 min in NuPage LDS buffer (Invitrogen, Carlsbad, CA, USA). All samples were resolved on a 4–12% SDS-PAGE gel and transferred to the Hybond ECL nitrocellulose filter (Sigma-Aldrich Corp., St. Louis, MO, USA). After blocking with Tris Buffered Saline with Tween (50 mM Tris-Cl PH7.6, 150 mM NaCl, and 0.05% Tween20) containing 3% BSA, the blots were incubated with mouse ant-GFP antibody (Takara Bio, Mountain View, CA, USA) at 1:4000 or mouse anti-myc (Invitrogen, Carlsbad, CA, USA) at 1:4000 overnight at 4 °C, followed by the horse raddish peroxidase (HRP)-conjugated anti-mouse IgG (Sigma-Aldrich Corp., St. Louis, MO, USA) at 1:4000 for 1.5 h at room temperature. The specific proteins were detected using ECL plus (Sigma-Aldrich Corp., St. Louis, MO, USA).

## 3. Results

### 3.1. Molecular Mapping of the t(2,8;q37,p11) Chromosomal Translocation Breakpoint in a Patient with Left-Sided Congenital Heart Defects

**Figure 1:**
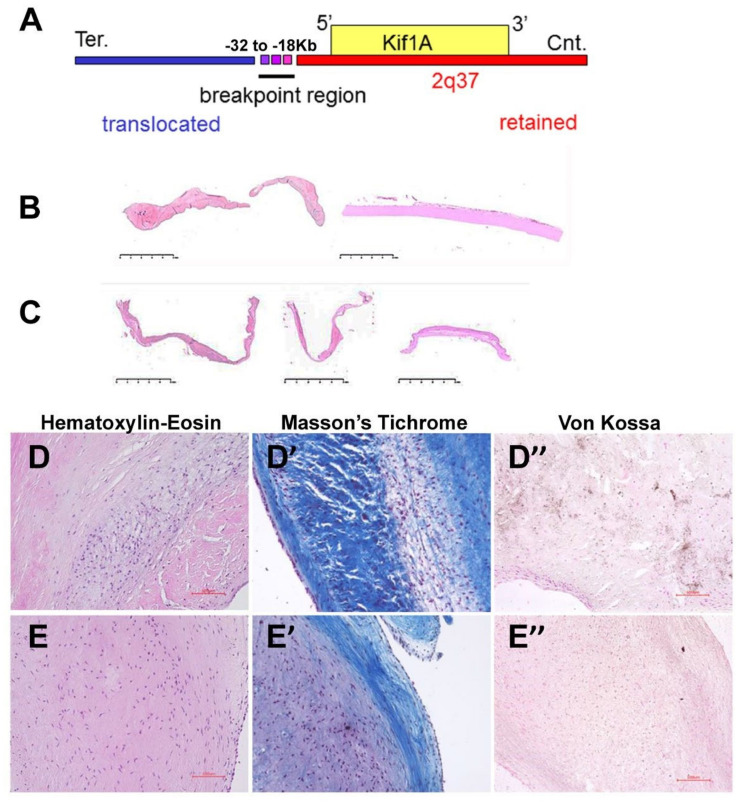
Patient with left-sided heart defects associated with a t(2,8;q37,p11) balanced translocation: (**A**) Molecular mapping of the 2q37 translocation breakpoint by fluorescence in situ hybridization. The transcription initiation site of the *KIF1A* gene is downstream of the breakpoint region. Whole mount, transverse sections of explanted aortic valve leaflets from (**B**) the patient and (**C**) an age- and sex-matched unaffected control. (**D**,**E**) Higher magnification images of sections from explanted aortic valve tissue from the patient (**D,D’’**) and an age and sex-matched unaffected control (**E**,**E’’**). D and E: Hematoxylin-Eosin stain showing interstitial cells; **D’** and **E’**: Masson’s Trichrome stain showing loss of interstitial cells accompanied by increased fibrosis (blue) in the patient; and **D’’** and **E’’**: Von Kossa stain showing calcification (brown) in the patient.

We identified a patient with left-sided heart defects in association with a balanced chromosome translocation (t(2,8;q37,p11). He was born with coarctation of the aorta and aortic valve stenosis. He underwent surgical repair for coarctation at age seven years in 1968, aortic valve replacement with a human homograft at age 29 years in 1990, and then again at age 49 years in 2009. He carries a balanced chromosomal translocation (t(2,8),2q37;8p11) (Figure 1A). Previous reports have associated left-sided heart defects with chromosomal deletions spanning 2q37, and consequently, we hypothesized that this patient’s 2q37 translocation breakpoint might affect the expression of a causal gene for left-sided heart defects. Therefore, in this study, we did not assess candidate genes at the 8p11 breakpoint. Toward that end, we performed molecular mapping of the 2q37 translocation breakpoint by fluorescence in situ hybridization on the patient, which localized the chromosome 2q37 breakpoint to a 14-kb region 18 kb upstream of the transcription initiation site of the *Kif1A* gene [18]. Furthermore, although there are other genes in the region of the 2q37 breakpoint, we focused on the *Kif1A* gene, given its proximity to the breakpoint. We obtained paraffin-embedded tissue from the patient’s explanted aortic valve from his second surgery in 1990 for analysis. The leaflets of the patient’s aortic valve were noticeably thickened (Figure 1B) compared to valve leaflet tissue from an age- and sex-matched normal control (Figure 1C). Histological analysis of the patient’s aortic valve leaflets revealed a dramatic loss of interstitial cells (Figure 1D compared to control in Figure 1E), replaced by diffuse fibrosis (Figure 1D’ compared to control in Figure 1E’) and severe calcification compared to the control (Figure 1D’’,E’’, respectively).

### 3.2. Kif1A Overexpression Impairs Cardiac Function

Recent studies have demonstrated that manipulations of human cardiac disease gene homologs in *Drosophila* can reproduce human cardiac phenotypes in the fly heart [14,19,20,21,22]. We reasoned that a breakpoint close to the *KIF1A* transcription start site might affect gene expression either by increasing or decreasing transcription. *Kif1A*is normally expressed in neuronal tissues in the mouse but has not been shown to be expressed in the heart [23]. We initially examined the effect of loss of Kif1A function on heart function in the adult fly using the Gal4-UAS system [24]. We used RNAi technology [17,24] to knockdown (KD) unc-104, the functional counterpart of *Kif1A* that exists as a single gene in the *Drosophila* genome. We examined heart function in mesoderm-specific unc-104 KD flies and in heterozygous deficiency flies (Df(2R)Exel6064/w1118, Df(2R)Exel7145/w1118) using high-speed video imaging of hearts in a semi-intact preparation [15]. We found that heart function was unaffected in either cardiac-specific KD or deficiency flies and was similar to that observed in laboratory wildtype flies with the matched genetic backgrounds (Supplementary Table S1), suggesting that unc-104 is not essential for normal heart development and adult function in the *Drosophila* system. This is also supported by the fact that normally unc-104 expression in the heart is almost undetectable (Supplementary Figure S1) and that no cardiac phenotype has been observed in *Kif1A* knockout mice [25].

**Figure 2:**
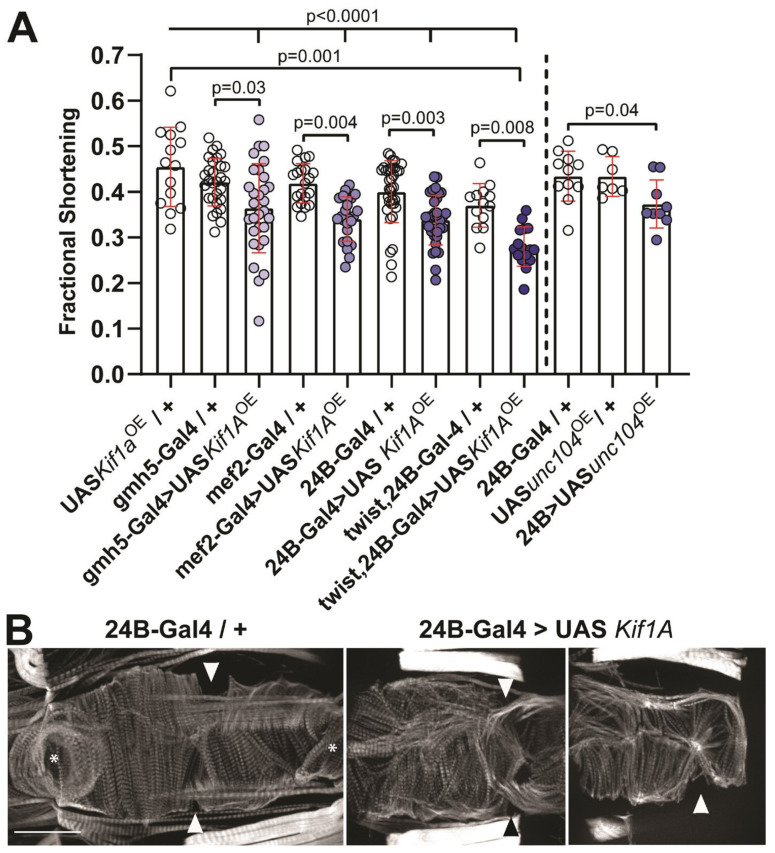
*Kif1A* overexpression causes reduced contractility and increased myofibrillar disarray: (**A**) Mesodermal (Twist;24B-Gal4 and 24B-Gal4, and mef2-Gal4) and cardiac-specific (GMH5-Gal4) drivers were used to drive (left) *Kif1A* and (right) unc104 overexpression in the hearts of 3-week-old flies. In all cases, overexpression of mouse *Kif1A* or fly unc104 significantly reduced fractional shortening, a measure of cardiac contractility, compared to controls. Significance was determined using a one-way analysis of variance and Tukey’s multiple comparisons post hoc test; number of flies in each group is shown in Supplementary Table S1. (**B**) Hearts from 3-week-old adult flies stained for F-actin: Control hearts (24B-Gal4/+) show compact myofibrillar structure and well-defined ostia (inflow tracts, arrowhead) and valve (asterisk). *Kif1A* overexpression resulted in hearts with primarily thin and disorganized myofibrils within the cardiac myocytes with many gaps due to myofibrillar disarray. For orientation purposes, the location of the inflow tracts is indicated by arrowheads. Scale bar is 50 microns; anterior is to the left.

We next tested the possibility that the breakpoint results in overexpression of *Kif1A* to affect heart function. We generated transgenic fly lines that overexpress the mouse Kif1A protein tagged with myc. Using the Gal4-UAS system, we drove overexpression specifically in mesoderm (using twist;24B-Gal4, 24B-Gal4, and mef2-Gal4) or specifically in myocardial cells (using GMH5-Gal4). Exogenous expression of mouse *Kif1A* was confirmed by Western blotting using anti-myc antibody (Supplementary Figure S2A). Hearts from 3-week-old *Kif1A* overexpressing flies demonstrated normal diastolic and systolic intervals (Supplementary Table S1), but in all cases, these hearts exhibited significant reductions in contractile function (measured as % fractional shortening, Figure 2A, Supplementary Table S1). Similarly, overexpression of the fly Kif1A, *unc104*, also reduced cardiac contractility (Figure 2A, right bars). Interestingly, in 1-week-old flies, contractility was not affected when *Kif1A* was overexpressed using a relatively weak cardiac-specific driver GMH5-Gal4 (Supplementary Table S1). However, by three weeks of age, cardiac contractility in these flies was strikingly compromised (Figure 2A), suggesting that *Kif1A* overexpression causes dose-dependent and progressive cardiac dysfunction with age. A dose dependency is also suggested by the increasingly severe reduction in fractional shortening as the strength of the driver increases (Figure 2A). For the strongest drivers used (24B-Gal4 and twist;24B-Gal4), this decreased contractility was correlated with an apparent dose-dependent increase in end systolic diameters (Supplementary Figure S3A) and less consistent decreases in the end diastolic diameters (Supplementary Figure S3B), indicating systolic dysfunction.

### 3.3. Kif1A Overexpression Impairs Cardiac Morphology

**Figure 3:**
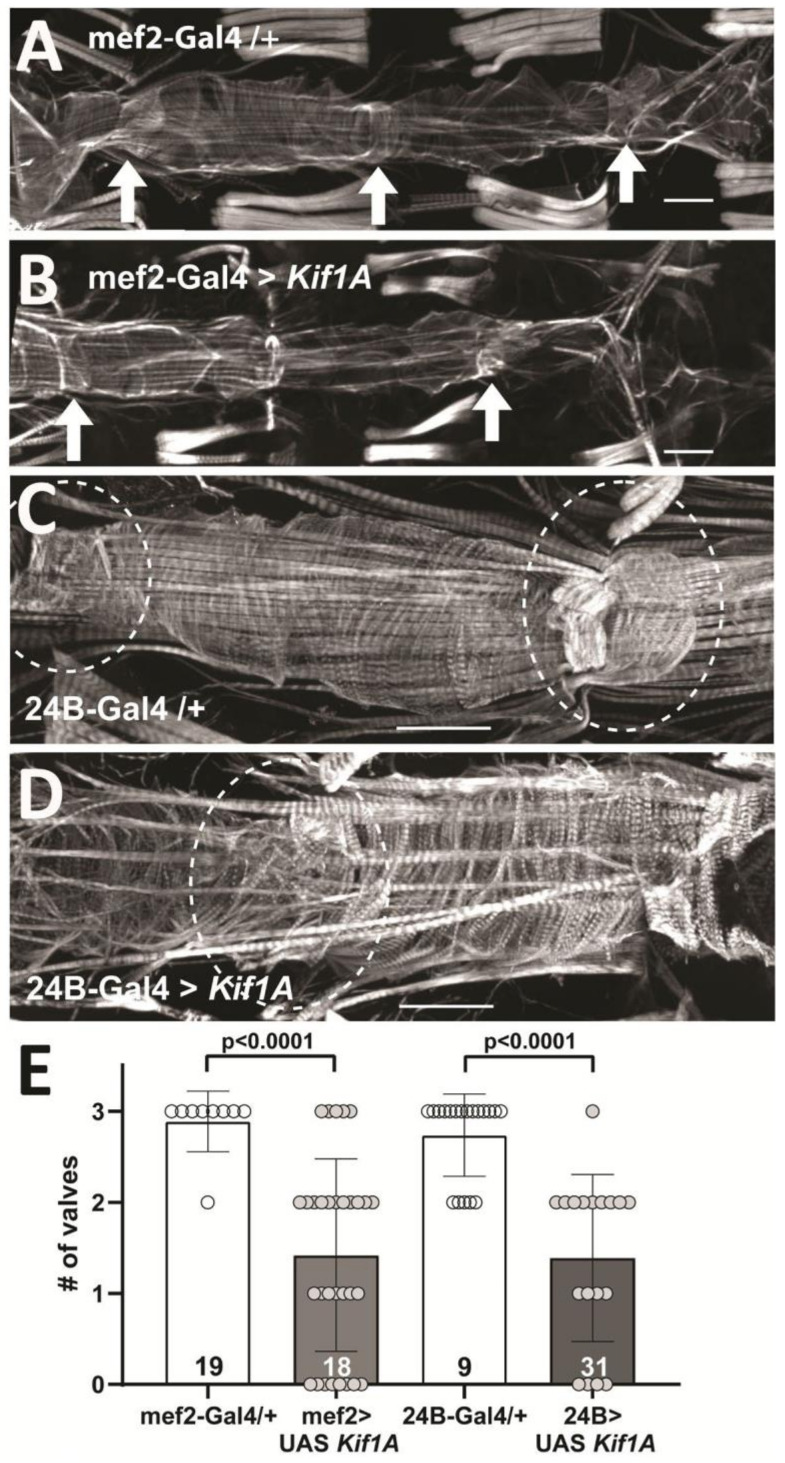
*Kif1A* overexpression causes valve remodeling: (**A**) F-actin staining with phalloidin of a control heart (mef2-Gal4/+) showing circumferential arrangement of myofibrils in the heart chambers and a denser myofibrillar arrangement in the 3 valve structures (indicated by arrows). Scale bar is 50 microns; anterior is to the left in all pictures. (**B**) In *Kif1A* overexpressing (OE) hearts (mef2-Gal4 > UAS *Kif1A*), the 2 identifiable valves (indicated by arrows) show reduced myofibrillar structure. (**C**) F-actin staining in a control heart (24B-Gal4/+) shows dense myofibrillar structure in the valve (dashed line circle). (**D**) Staining in *Kif1A* OE hearts (24B-Gal4 > UAS*Kif1A* OE) shows disorganized and loosely arranged myofibrils in the valve. Note overall reduction in F-actin containing myofibrils in *Kif1A* OE hearts in both Figure 3B,D. (**E**) Quantitation of identifiable valves in controls (24B-Gal4/+ and mef2-Gal4/+) and in F-actin stained hearts from *Kif1A* OE flies (24B > UAS *KifAa* and mef2 > UAS *Kif1A*): The number of hearts is indicated in each bar; ****p < 0.0001, Mann–Whitney test.

We examined the morphology of adult hearts with *Kif1A* overexpression. Hearts in adult *Drosophila* are located just below the cuticle on the dorsal side of the abdomen and are composed of three distinguishable muscles: circumferential muscles, longitudinal muscles, and alary muscles [26,27]. The circumferential muscles are the working myocardial cells. Hearts were stained with fluorescent phalloidin to visualize the filamentous actin (F-actin) structure in the myofibrils. In controls, the myofibrils are circumferentially arranged to facilitate squeezing of the heart tube and hemolymph ejection (Figure 2B, 24B-Gal4/+). In hearts from all *Kif1A* overexpressing flies, myofibrils were not tightly packed, exhibited significant disarray within the myocardial cells (Figure 2B, 24B-Gal4>*Kif1A*, see also Figure 3), and were consistent with the observed reductions in contractility.

Using SDS-PAGE electrophoresis, we confirmed a significant reduction in myosin heavy chain protein in isolated hearts overexpressing *Kif1A* (Hand-Gal4 > UAS-*Kif1A*) compared to controls (Supplementary Figure S2B). These findings indicate that *Kif1A* overexpression disrupts the myofibrillar structure and reduces cardiac muscle protein content.

In addition, we noted that hearts overexpressing *Kif1A* also exhibited valve dysfunction compared to controls (Supplementary Video S1). The linear fly heart is normally divided into four chambers by the presence of three internal valves with valve cellular structure characterized by a dense arrangement of myofibrils (Figure 3A, arrows). However, in flies where *Kif1A* is overexpressed in the heart, some valves appeared to be absent (Figure 3B, Supplementary Video S1, bottom two hearts). In addition, the arrangement of myofibrils in the valves was disorganized and diffuse (compare Figure 3C,D). Quantitation of identifiable valves from phalloidin stained preparations showed a significant reduction in the number of well-defined valves in *Kif1A* overexpressing flies compared to controls (Figure 3E).

**Figure 4:**
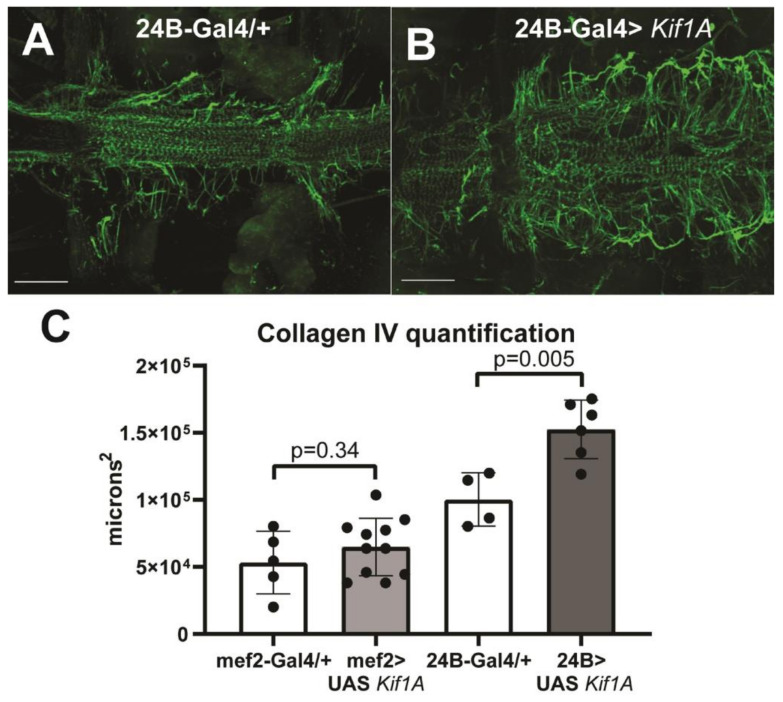
*Kif1A* overexpression increases collagen IV deposition: (**A**) Anti-pericardin (collagen IV homolog) staining reveals the extracellular matrix structure surrounding the cardiac tube in control flies expressing the muscle cell driver 24B-Gal4. (**B**) Muscle-specific *Kif1A* overexpression caused a significant increase in the extent of cardiac-associated collagen IV network. Scale bar is 50 microns; anterior is to the left. (**C**) Quantification of the ECM networks from Z-stacks shows significant increases in response to muscle cell-specific *Kif1A* OE. Significance was determined by two-tail, unpaired T-tests.

We also examined the presence and arrangement of the extracellular collagen network that normally surrounds the fly heart. One of the primary components of this network is pericardin, a type IV collagen, which can be visualized by anti-pericardin staining revealing a “fishnet” arrangement of collagen fibers that surrounds and supports the heart tube. Hearts overexpressing *Kif1A* exhibited an increase in the number and thickness of collagen fibers (Figure 4A–C). Quantification of the area of collagen surrounding the heart showed a significant increase in hearts where *Kif1A* was overexpressed using the stronger 24B muscle driver, again suggesting a dose-dependent effect (Figure 4C).

### 3.4. Kif1A Overexpression Does Not Affect Early Cardiac Development

To test whether the effect of *Kif1A* overexpression on heart function/morphology occurred during *Drosophila* embryological heart development, we analyzed the cardiac precursor cell number and the distribution of the cell polarity marker Dlg (disk large), which is normally localized to the dorsal-lateral sides of the heart. Analysis of stage 18 embryos revealed no differences in overall cell number and in the Dlg localization between wild-type and *Kif1A* overexpressing stage 18 embryos (Supplementary Figure S4), indicating that overexpression of *Kif1A* does not affect cardiomyocyte commitment or cell polarity in early stages of *Drosophila* development.

### 3.5. Overexpression of Kif1A Affects Actin Fiber Distribution and Mammalian Cell Morphology

**Figure 5:**
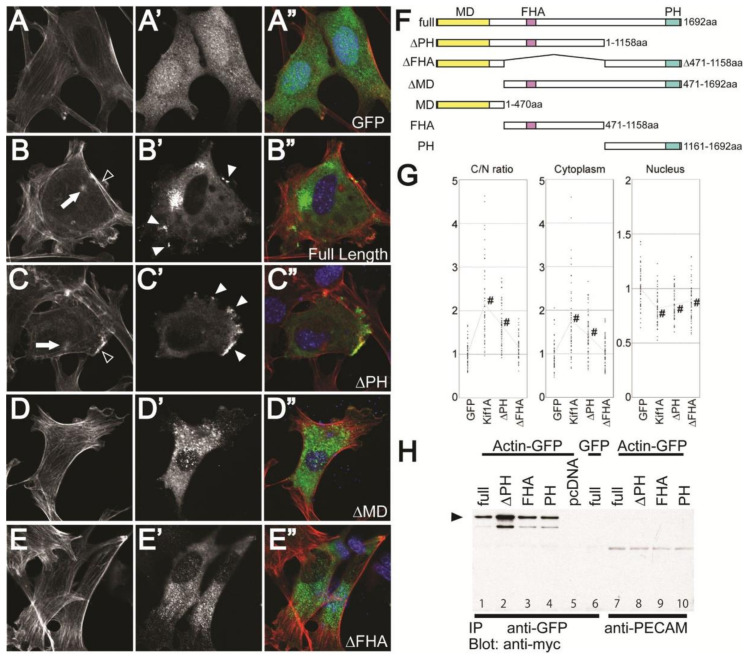
Actin cytoskeleton is affected in *Kif1A*-transfected C2C12 myoblasts: (**A**–**E**) Phalloidin staining reveals F-actin organization in transfected C2C12 cells. α−GFP or α−myc staining identifies cells transfected with (**A’**) AcGFP (controls), (**B’**) full-length myc-tagged *Kif1A*, (**C’**) myc-tagged *Kif1A* lacking the PH domain, (**D’**) myc-tagged *Kif1A* lacking the MD domain, and (**E’**) myc-tagged *Kif1A* lacking the FHA domain. The F-actin organization is displaced more towards the periphery of the cell (open arrowheads) where there are aggregates of *Kif1A* (solid arrowheads), as shown in Figure 5B,C. This effect is observed in the construct lacking the lacking the PH domain but not in those lacking the motor domain or the forkhead-associated domain, implying that normal motor domain function is required for overexpressed Kif1A protein to exert its effect. (**F**) Schematic illustrating the structure of full-length and truncated Kif1A proteins. MD: kinesin motor domain, FHA: forkhead associated domain, and PH: Pleckstrin homology domain. All the constructs listed in Figure 5F were tagged with myc for detection; (**G**) *Kif1a* transfection increases cell size. Two-dimensional cell surface area was measured in C2C12 cells transfected with AcGFP, full-length *Kif1A*, truncated *Kif1A* ∆FHA, or ∆PH. The area occupied by cytoplasm in *Kif1A*-transfected cells is significantly larger than control (Ac-GFP). Numbers of cells analyzed: GFP = 54, *Kif1A* = 51, ∆PH = 53, and ∆FHA = 52; non-repeated measures ANOVA followed by Student–Newman–Keuls post hoc test. #*p* < 0.01 compared to GFP-transfected cells. (**H**) Lysate from C2C12 cells co-transfected with actin-GFP and full-length (full) or truncated *Kif1A* (∆PH, FHA, and PH) were immunoprecipitated with anti-myc or anti-PECAM as a negative control. Actin was detected (arrowhead) by anti-GFP antibodies in all the samples including cells transfected with truncated *Kif1A*. Inputs are shown in Supplementary Figure S7.

Based on our results in the fly model, we aimed to determine if overexpression of *Kif1A* also affects morphology in mammalian muscle cells. *Kif1A* contains three known domains: the kinesin motor domain (MD), the forkhead associated domain (FHA), and the Pleckstrin homology domain (PH) (Figure 5F). In neurons, the MD interacts with and moves anterograde along microtubules using energy from microtubule-stimulated ATP hydrolysis. The FHA domain plays an important role in stabilizing the monomeric form of the Kif1A protein, which is critical for Kif1A motor activity [28]. The PH domain is known to physically associate with the lipid membrane of neurotransmitter vesicles [29].

In order to test which domain contributes to the altered myoblast cell morphology, we constructed a series of truncated Kif1A proteins (Figure 5F) and overexpressed them in C2C12 myoblast cells by transfection with a mouse *Kif1A* expression construct. Control cells, transfected with GFP, exhibited rectangular, extended shapes with many mostly longitudinally oriented actin filaments (Figure 5A–A”). Cells overexpressing full-length *Kif1A* or *Kif1A* lacking the C terminal PH domain (∆PH, see Figure 5F for domain structure and truncated constructs) assumed broader, more expanded shapes (Figure 5B–B”,C–C”). The F-actin staining shows a rearrangement of stress fibers to the periphery in *Kif1A* overexpressing cells (Figure 5B, open arrowhead). In addition, Kif1A protein (detected by the myc tag) in these overexpressing cells appeared to be unevenly distributed, forming isolated aggregations and localized densities (Figure 5B’,C’, solid arrowheads). Interestingly, the large regions of F-actin displacement appear to be coincident with the larger Kif1A aggregations (Figure 5B”), and peripheral F-actin accumulations (Figure 5B’,C’, open arrowheads) are coincident with the localized densities (Figure 5B’,C’ solid arrowhead). In contrast, cells transfected with *Kif1A* lacking the MD or FHA domains showed distributions of actin filaments that were similar to control cells (Figure 5D–D”,E–E”) and the myc-tagged Kif1A protein was evenly distributed throughout the cells.

Expression of the MD, FHA, or PH domains by themselves does not produce any changes in cell morphology or actin fiber formation (Supplementary Figure S5). Interestingly, expression of MD alone results in the nuclear localization of this truncated protein (Supplementary Figure S6A’). It is likely that the FHA domain is responsible for the cytoplasmic localization of *Kif1A* since the ∆PH construct (MD-FHA combination) is not found in the nucleus (Figure 5C”).

To quantify an effect of *Kif1A* OE on cell morphology, we measured two-dimensional cell size normalized to the size of the nucleus. *Kif1A* overexpression resulted in a dramatic increased in size compared to GFP-transfected control cells 36 h after transfection (Figure 5G), suggesting that overexpression of *Kif1A* alters normal cell morphology. Overexpression of *Kif1A* that lacks the PH domain (∆PH) resulted in a slight increase in cell size compared to GFP-expressing controls (Figure 5C–C”,G) but this increase was smaller than that observed following overexpression of the full-length protein. Overexpression of *Kif1A* lacking either the FHA domain (∆FHA) had little effect on cell size (Figure 5G), which was consistent with the lack of effect on morphology (Figure 5E–E”). Together, these data suggest that all three functional domains are required for the significantly increased cell size seen in response to overexpression of the full-length *Kif1A*.

**Figure 6:**
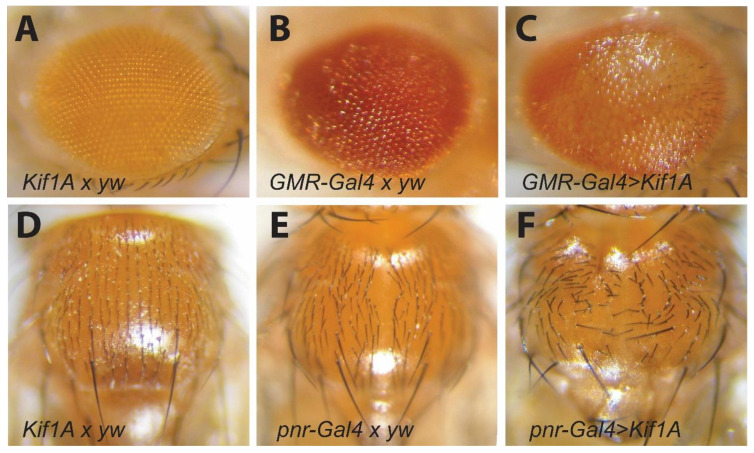
*Kif1A* overexpression impairs viability and cell polarity: *Kif1A* overexpression impairs viability of ommatidia and pigment cells. (**A**) One-week-old *Kif1A* x yw and (**B**) GMR-Gal4xyw controls. (**C**) Driven *Kif1A* OE in age-matched flies exhibited rougher and more decolorized external eyes compared to genetic controls. *Kif1A* overexpression impairs bristle arrangement. (**D**) One-week-old *Kif1A* x yw and (**E**) pnr x yw controls. (**F**) Overexpression of *Kif1A* in dorsal mesoderm using the Pnr-Gal4 driver resulted in disorientated bristle arrangement compared to genetic controls.

We also tested the ability of Kif1A to directly bind actin. HEK293T cells were co-transfected with full-length or truncated myc-tagged *Kif1A* and GFP-tagged actin. Immunoprecipitation with anti-GFP antibodies pulled down full-length and all truncated forms of the myc-tagged *Kif1A* (Figure 5H and Supplementary Figure S7). Note that ∆PH associates with G-actin more strongly than the other truncated *Kif1A* constructs, consistent with the stronger accumulation of ∆PH at the edge of C2C12 cells (see Figure 6C). Taken together, these results suggest that Kif1A affects actin filament organization and mammalian cell morphology.

### 3.6. Kif1A OE Affects Cell Viability and Cell Polarity

We then determined whether the reorganization of the cellular actin cytoskeleton in both fly hearts and in cultured cardiomyocytes might also affect cell polarity. We took advantage of two model systems in the fly that provide readouts for alterations in cell polarity: ommatidium development in the fly eye [30] and bristle pattern formation in the thorax [31]. We overexpressed *Kif1A* specifically in photoreceptors and pigment cells in developing eyes using the GMR-Gal4 driver [32]. Eyes in *Kif1A*/yw outcrossed controls show the normal ommatidium arrangement with the expected white eye phenotype (Figure 6A). GMR-Gal4/yw controls show the expected red eye phenotype but are slightly glassy in appearance, likely the result of some developmental effects due to overexpression of Gal4 itself from this strong driver line (Figure 6B). In contrast, eyes from *Kif1Aa* OE flies exhibited an edematous, glassy-eye appearance and disorganized cell arrangement (Figure 6C compared to Figure 6A,B). These phenotypes, along with the reduced red eye color, suggest a reduction in overall cell viability. We also overexpressed *Kif1A* in the dorsal part of the embryo using the Pannier (Pnr)-Gal4 driver [33]. Again, we observed the normal, regular arrangement of bristles in *KIF1A*/yw controls (Figure 6D) and a slight disruption of bristle orientation in the Pnr-Gal4/yw controls that may reflect Gal4 leakiness from this driver [34] (Figure 6E). However, bristle orientation in the Pnr > *Kif1a* flies was severely disrupted, indicating that *Kif1Aa* overexpression also compromises cell polarity (Figure 5F).

### 3.7. Overexpression of Kif1A Inhibits Muscle Maturation

**Figure 7:**
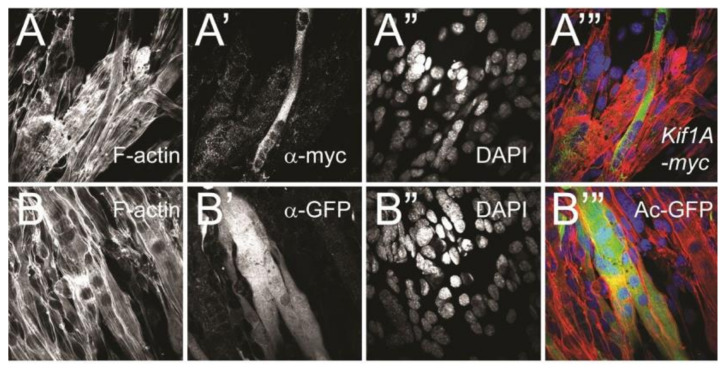
Kif1A overexpression affects muscle maturation: Twenty-four hours after transfection (with Kif1A-myc or AcGFP), C2C12 myoblast cells were switched to differentiation medium for 36 h and then fixed and stained. (**A–A’’’**) Kif1A transfected cells. (**B–B’’**) GFP-transfected cells. (**A**,**B**) Phalloidin staining reveals F-actin organization in all cells. (**A’**) Anti-myc staining to detect Kif1A expressing cells and (**B’**) anti-GFP staining to detect GFP-transfected control cells. (**A”**,**B”**) DAPI nuclear staining. (**A’’’**,**B’’’**) Merged images: note that differentiated C2C12 cells with Kif1A overexpression (**A**,**C–C’”**) formed a syncytium but contained scant cytoplasm.

Embryonic analysis of cardiac precursor cells in mesodermal *Kif1A* overexpressing flies indicates that *Kif1A* does not affect the cardiac cell commitment (Supplementary Figure S3). However, because Kif1A altered actin organization in myoblast cells (Figure 5), we hypothesized that Kif1A may affect or redirect actin remodeling that occurs during myocyte maturation. To test this hypothesis, we initiated differentiation of the *Kif1A*-transfected C2C12 cells into myocytes in vitro (Figure 7). Cells transfected with the GFP control vector formed syncytia with abundant muscle myofibrils (Figure 7B). *Kif1A*-transfected cells also formed syncytia; however, they had very scant cytoplasm (Figure 7A”’). Quantification of the area of transfected cells reveals a significantly smaller total area per field of view and a smaller number of associated nuclei for cells transfected with *Kif1A* compared to GFP-transfected controls (Supplementary Figure S8). Interestingly, the ratio of cell area per nucleus was the same for both GFP and *Kif1A*-transfected cells. Taken together, these results suggest that overexpression of *Kif1A* impairs myocyte maturation, likely through disruption of actin organization, which is consistent with the effects of *Kif1A* overexpression in the fly heart.

Previous studies have demonstrated that the Kif1A protein directly interacts with tubulin in microtubules [23,35]. Therefore, we examined the distribution and morphology of actin and microtubules (tubulin) in C2C12 cells where *Kif1A* was overexpressed. We found no apparent morphological changes in size and distribution of actin (Supplementary Figure S5) or tubulin (Supplementary Figure S6) when *Kif1A* is overexpressed. This suggests that excess levels of *Kif1A* impair cardiac development primarily due to its effect on actin organization

## 4. Discussion

In this study, we identify the *Kif1A* gene as a candidate gene for causing left-sided heart defects and utilize *Drosophila* to demonstrate the pathologic effects of overexpression of the *Kif1A* gene on heart development and function. We demonstrate that, while KD of *Kif1A* had no effect on heart development or function, ectopic expression of *Kif1A* in the *Drosophila* heart causes dysplastic valves and cardiac remodeling associated with diminished contractile capability (Figure 2 and Figure 3). Interestingly, these observations are among the hallmark features of one of the most severe human congenital heart defects, hypoplastic left heart syndrome (HLHS). Although the balanced translocation in this patient with left-sided heart defects may be a coincidental finding, the observation in this study that overexpression of *Kif1A* in *Drosophila* phenocopies at least one of the primary cardiac defects in the patient, i.e., aortic valve disease, is compelling.

Although disease-associated mutations often cause loss of function, mutations causing overexpression of the regulator of calcineurin have previously been linked to sporadic congenital heart disease [30] and overexpression of limb-bud and heart, a regulator of transcription, in the mouse heart recapitulates CHD reported in humans with partial trisomy 2p syndrome [36]. Consistent with previous findings in neural cells [16,37], our studies also indicate that exogenously expressed Kif1A protein accumulates at the edge of muscle cells. Our cell culture experiments suggest that this effect requires the MD and FHA domains of the Kif1A protein. The requirement for MD (Figure 5) suggests that the effect is exerted as the Kif1A protein translocates along microtubules. Combined expression of the MD together with FHA domain (∆PH) caused accumulation of protein at the leading edge of the cell, suggesting that it is the pH domain that normally prevents this Kif1A accumulation (Figure 5C’). A possible link between Kif1A and actin organization may lie in Kif1A’s ability to interact directly with actin (Supplementary Figure S8). Kif1A mislocalization/aggregation may sequester G-actin and interfere with F-actin formation and organization. Previous studies have demonstrated that actin mutations causing malformation of the cytoskeleton lead to the development of cardiomyopathies [38,39]. Recently, mutations in the alpha cardiac actin gene were identified in association with familial atrial septal defects [38], suggesting an important role for cardiac actin in cardiac development. Our studies demonstrate that overexpression of *Kif1A* during cardiac development results in structural heart defects, likely by affecting actin organization.

### Pathological Effects Associated with Kif1A Overexpression in the Heart

Based on the identification of a balanced translocation near *Kif1A* in a patient with left-sided heart defects, we used the *Drosophila* heart model to manipulate Kif1A protein levels. We found that overexpression of Kif1A in cardiomyocytes in the fly had a striking effect on the myofibrillar structure, cardiac contractility, fibrosis, and valve differentiation. These data are strikingly consistent with the histopathology in the patient’s valve abnormalities and fibrosis (Figure 1), as well as what has been described in a subset of human HLHS hearts [5,40,41].

In summary, we have identified a patient with left-sided heart defects in association with a t(2;8)(q37;p11) balanced translocation. Molecular mapping of the 2q37 translocation breakpoint, a locus previously associated with left-sided obstructive lesions, implicated the *KIF1A* gene as a candidate gene for causing these defects. In this study, we demonstrated that overexpression of *Kif1A* in the *Drosophila* heart caused morphological changes reminiscent of those that occur in HLHS. Specifically, our findings suggest that disruption of the F-actin organization causes ventricular hypoplasia with decreased function due to a primary defect in ventricular development in addition to an accompanying defect in valve development. Future studies in mammalian animal model systems should yield important new insights into the role of the F-actin organization in causing left-sided heart defects.

## Supplementary Materials

The following are available online at https://www.mdpi.com/2308-3425/7/2/22/s1, Supplementary Table S1. Quantification of function in hearts from control and *Kif1A* overexpression and knockdown, Supplementary Figure S1. Unc expression, Supplementary Figure S2. *Kif1A* overexpression causes systolic dysfunction, Supplementary Figure S3. Western Blot analysis, Supplementary Figure S4. Cardiac primordial cell arrangement is not affected during embryogenesis, Supplementary Figure S5. Actin cytoskeleton is not affected by overexpression of the individual *Kif1A* functional domains, Supplementary Figure S6. Microtubule structure is unaffected by *Kif1A* overexpression, Supplementary Figure S7. *Kif1A* physically associates with G-actin in myocytes, Supplementary Figure S8. Quantification of *Kif1A*-transfected C2C12 induced myocytes, Video S1. High-speed movies of in situ *Drosophila* hearts.
