# Supplementary material for: Overexpression of Kif1A in the Developing Drosophila Heart Causes Valvar and Contractility Defects: Implications for Human Congenital Heart Disease
Source: J Cardiovasc Dev Dis. 2020 Jun 2;7(2):22. doi: 10.3390/jcdd7020022 (PMC7345553; doi:10.3390/jcdd7020022)
Supplement: Supplementary file 1 [file jcdd-07-00022-s001.zip › jcdd-818444 SF.docx]

Supplementary Materials

Overexpression of *Kif1A* in the Developing *Drosophila* Heart Causes Valvar and Contractility Defects: Implications for Human Congenital Heart Disease

Takeshi Akasaka ^1,3,†^, Karen Ocorr ^1,†,^*, Lizhu Lin ^2^, Georg Vogler ^1^, Rolf Bodmer ^1^ and Paul Grossfeld ^2,^*

^1^ Development, Aging & Regeneration Program, Sanford Burnham Prebys Medical Discovery Institute, La Jolla, California, USA 92037; akasaka-cba@umin.ac.jp (T.A.); gvogler@sbpdiscovery.org (G.V.); rolf@sbpdiscovery.org (R.B.)

^2^ Division of Cardiology, Department of Pediatrics, UCSD School of Medicine, San Diego, California, USA; lilin@health.ucsd.edu (L.L)

^3^ Cardiology and Catheterization Laboratories, Shonan Fujisawa Tokushukai Hospital, Fujisawa City, 251-0041 Japan ; akasaka-cba@umin.ac.jp (T.A.)

**†** These authors contributed equally to this manuscript

***** Correspondence: pgrossfeld@ucsd.edu (P.G.); kocorr@sbpdiscovery.org (K.O.); Tel. 858-966-5855 (P.G.); Tel. 858-795-5125 (K.O.)

**Table S1.** Quantification of function in hearts from control and *Kif1A* over-expression and knockdown (KD) flies.

| **Genotype** | **Age (weeks)** | **N** | **Heart Period (sec)** | **Diastolic Interval (sec)** | **Systolic Interval (sec)** | **Diastolic Diameter (microns)** | **Systolic Diamter (microns)** | **Fractional Shortening** |
| --- | --- | --- | --- | --- | --- | --- | --- | --- |
| **UAS-Kif3 x yw (control)** | 1 | 13 | 0.62 ± .04 | 0.41 ± .04 | 0.21 ± .01 | 75.4 ± 2.4 | 46.9 ± 2.4 | 0.38 ± .02 |
|  | 3 | 16 | 0.75 ± .06 | 0.51 ± .06 | 0.24 ± .01 | 74.8 ± 2.4 | 45.5 ± 2.4 | 0.39 ± .02 |
| **twist24B-Gal4 x yw (control)** | 1 | 14 | 0.80 ± .06 | 0.57 ± .06 | 0.22 ± .01 | 68.6 ± 2.9 | 43.6 ± 2.7 | 0.37 ± .02 |
|  | 3 | 15 | 0.75 ± .06 | 0.51 ± .06 | 0.24 ± .01 | 69.3 ± 3.8 | 43.6 ± 2.7 | 0.35 ± .02 |
| **GMH5 x yw (control)** | 1 | 8 | 0.63 ± .05 | 0.40 ± .05 | 0.23 ± .02 | 73.2 ± 3.9 | 46.1 ± 2.6 | 0.37 ± .02 |
|  | 3 | 29 | 0.70 ± .03 | 0.47 ± .03 | 0.23 ± .01 | 83.9 ± 1.9 | 48.4 ± 1.2 | 0.42 ± .01 |
| **UAS-Kif3 x twist24B-Gal4** | 1 | 15 | 0.74 ± .07 | 0.51 ± .07 | 0.22 ± .01 | 74.3 ± 3.0 | 53.0 ± 3.3 | 0.29 ± .02 |
|  | 3 | 16 | 0.72 ± .06 | 0.47 ± .04 | 0.26 ± .03 | 69.7 ± 1.8 | 50.3 ± 1.6 | 0.28 ± .01 |
| **UAS-Kif3 x GMH5-Gal4** | 1 | 15 | 0.75 ± .05 | 0.53 ± .04 | 0.23 ± .01 | 76.3 ± 2.4 | 47.1 ± 1.7 | 0.38 ± .02 |
|  | 3 | 15 | 0.78 ± .07 | 0.51 ± .05 | 0.27 ± .02 | 73.2 ± 3.3 | 48.7 ± 2.6 | 0.33 ± .02 |
| **24B-Gal4 x w^1118^ (control)** | 1 | 13 | 0.77 ± .1 | 0.57 ± .09 | 0.21 ± .01 | 101.7 ± .2.7 | 57.6 ± 2.6 | 0.43 ± .02 |
| **24B-Gal4 x UAS-unc104a** | 1 | 16 | 0.77 ± .06 | 0.58 ± .06 | 0.19 ± .004 | 88.5 ± 2.8 | 50.2 ± 2.0 | 0.43 ± .01 |
| **24B-Gal4 x UAS-unc104b** | 1 | 17 | 0.64 ± 0.09 | 0.44 ± .09 | 0.20 ± .007 | 95.6 ± 3.4 | 57.2 ± 2.9 | 0.41 ± .01 |
| **24B-Gal4 x w^1118^ (control)** | 3 | 35 | 0.81 ± .05 | 0.57 ± .06 | 0.24 ± .01 | 102.9 ± 1.7 | 61.9 ± 1.7 | 0.40 ± .01 |
| **24B-Gal4 x UAS-kif3** | 3 | 36 | 0.85 ± 0.04 | 0.62 ± .04 | 0.23 ± .01 | 101.7 ± 2.0 | 67.4 ± 1.8 | 0.34 ± .01 |
| **mef2-Gal4 x w^1118^ (control)** | 3 | 20 | 0.91 ± .07 | 0.65 ± .07 | 0.27 ± .01 | 94.9 ± 2.4 | 55.2 ± 1.6 | 0.42 ± .01 |
| **mef2-Gal4 x UAS-kif3** | 3 | 25 | 0.94 ± 0.11 | 0.63 ± .09 | 0.30 ± .02 | 87.6 ± 2.1 | 57.8 ± 1.6 | 0.34 ± .01 |

Over-expression of Kif1A or the fly homolog unc104was induced using four different tissue-specific drivers: twist;24B-Gal4 (strong mesoderm-specific driver), 24B-Gal4 and mef2-Gal4 (relatively weaker mesoderm-specific drivers) and GMH5 (cardiac-specific drivers) and indicated cardiac parameters were measured. KD of Kif1A was achieved by crossing flies with the mesoderm-specific driver 24B-Gal4 with flies containing UAS-Kif1A RNAi constructs and in flies heterozygous for deletions that span the Kif1A locus (deficiency lines, Df). Data is shown as Mean ± SEM; significance was determined using One-way ANOVA or unpaired t-tests; **p* < 0.5, ***p* < 0.01, ****p* < 0.001.


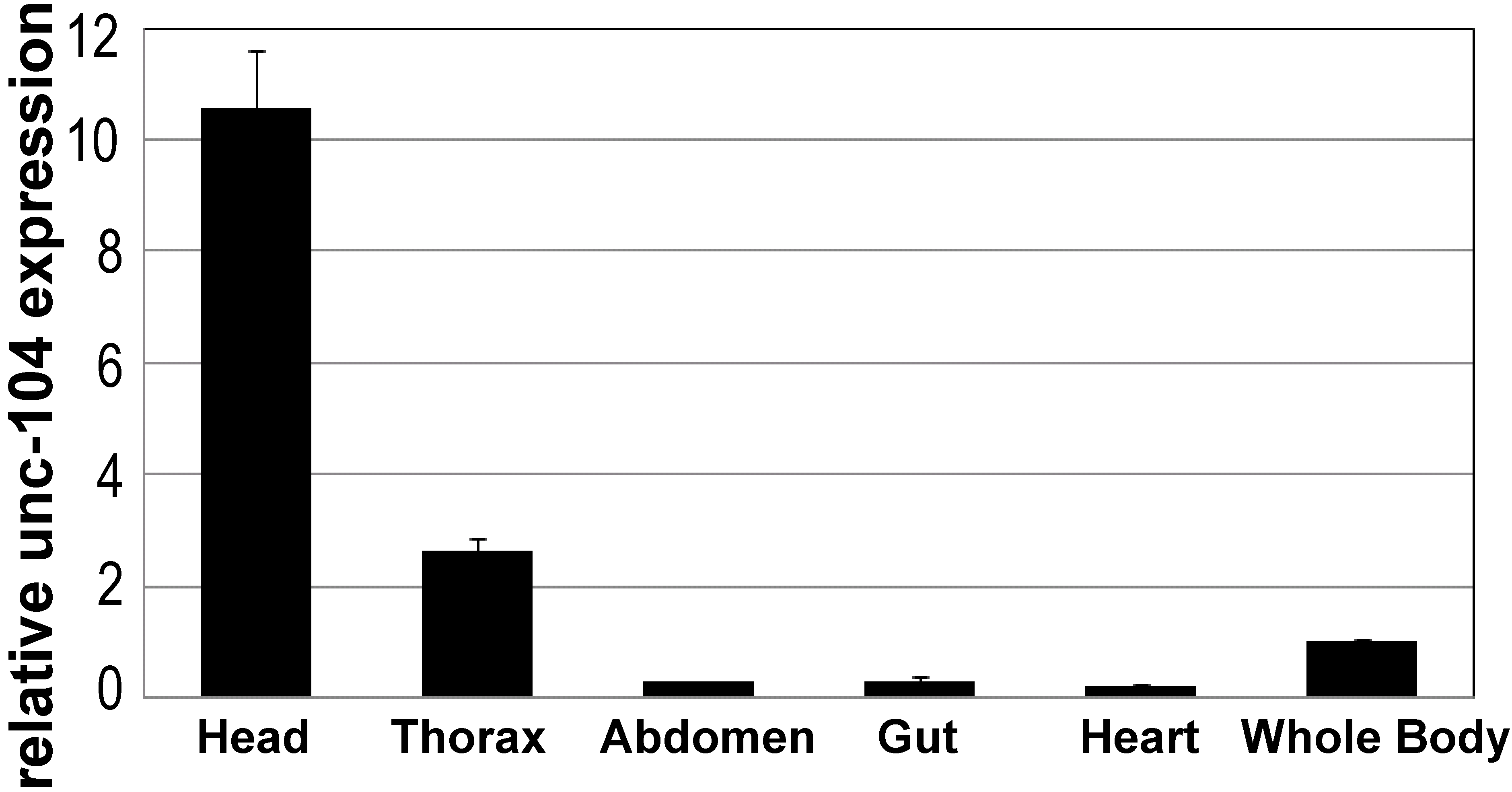


**Figure S1.** (**A**) Relative expression of unc-104 (*KIF1A* homologue) in different tissues from 1-week-old adults (normalized to actin expression). Unc-104 expression was enriched 10-fold in the head and 3fold in thorax (compared to whole body), but its wildtype expression was significantly lower in the heart (18% compared to whole body). (**B**) End Systolic Diameters (ESD) from the same hearts shown in (**A**) are significantly increased compared to controls in hearts where stronger drivers (24B-Gal4 and twist, 24B-Gal4) were used to overexpress *Kif1A*/unc104.


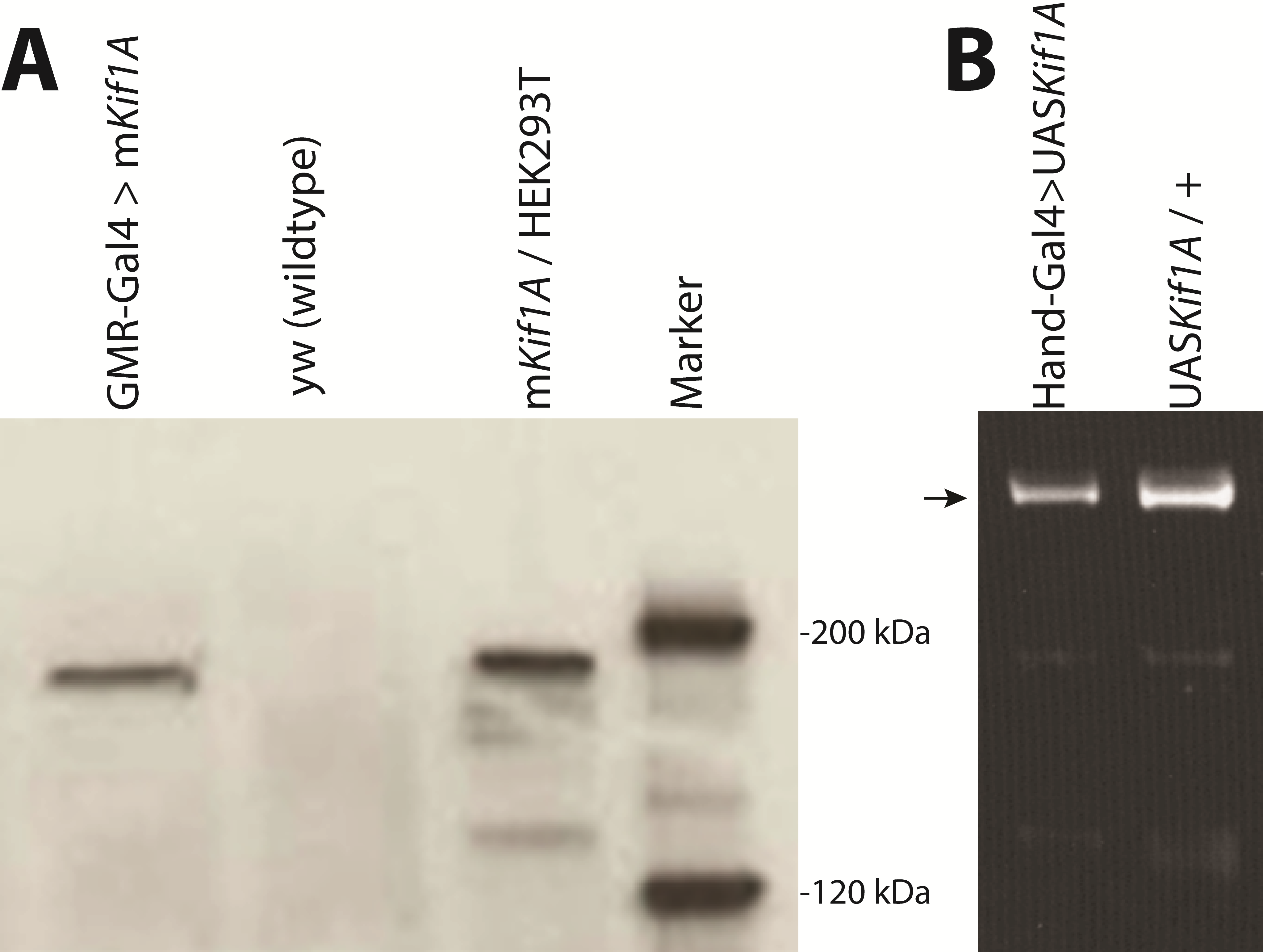


**Figure S2.** (**A**) Western Blot Analysis confirms *Kif1A* over-expression (OE). Expression of the myc tagged mouse Kif1A protein driven by the GMR-Gla4 was detected with anti-myc antibodies in flies that also had the GMR-Gal4 driver but not in control flies (yw). Kif1A protein was also detectable in transfected HEK293T cells. (**B**) Myosin heavy chain (MHC) is reduced in *Kif1A* OE hearts. Hearts were homogenized and proteins separated by SDS PAGE (5 hearts per lane). Proteins were detected with Coomassie Fluor Orange (Thermo Fisher Scientific), showing MHC, which is the most prominent protein in muscle tissue (arrow).


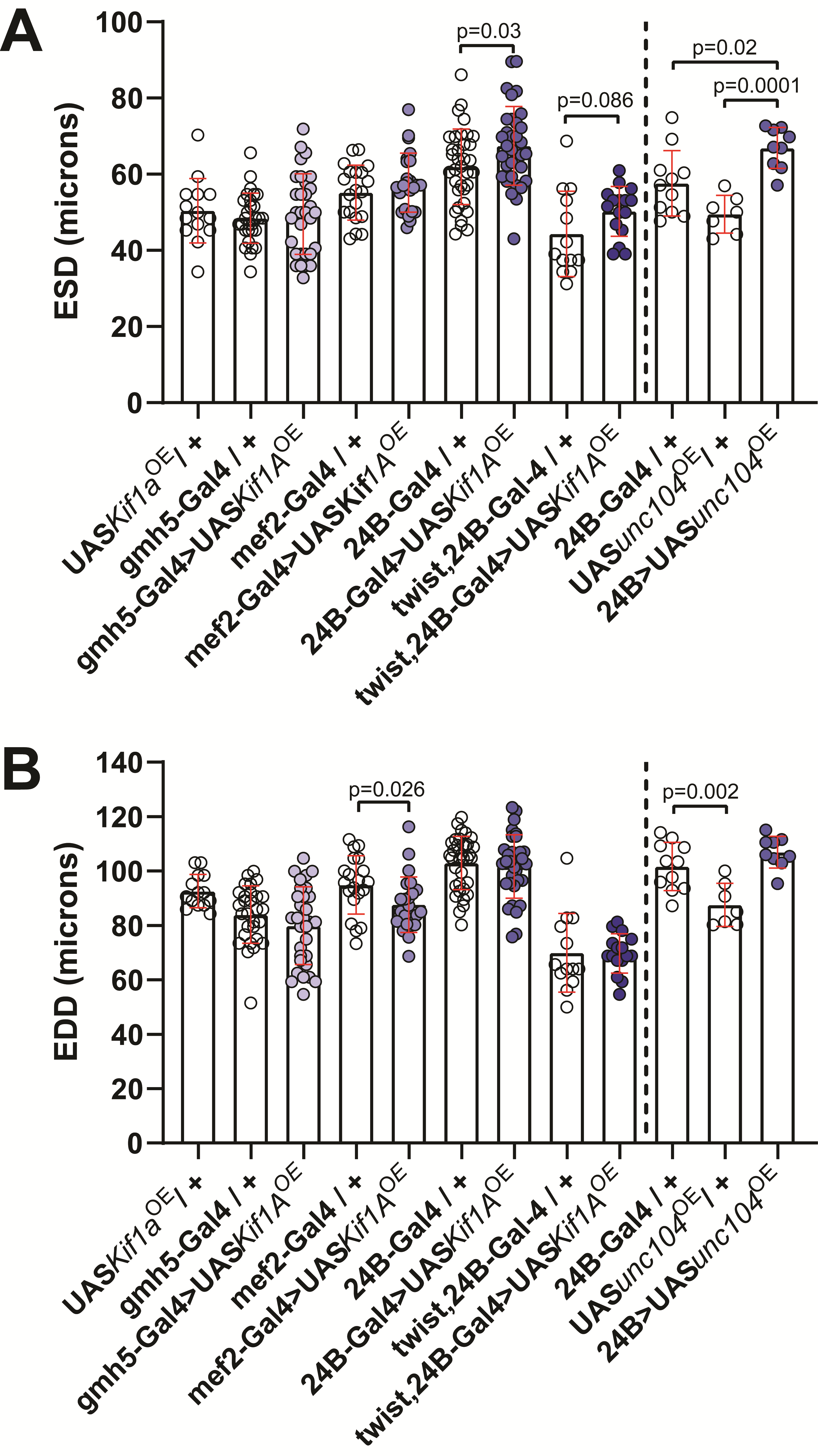


**Figure S3.** *Kif1A* overexpression causes systolic dysfunction (**A**) End Systolic Diameters (ESD) from the hearts shown in text Figure 2 are significantly increased compared to controls in hearts where stronger drivers (24B-Gal4 and twist, 24B-Gal4) were used to overexpress *Kif1A*/unc104. (**B**) There were minimal effects of *Kif1A* OE on End Diastolic Diameters (EDD). Because the EDD varied significantly between flies with different genetic backgrounds, significance was determined by unpaired two-tail, t-tests between the tissue-specific driver controls and the corresponding OE flies. A one-way ANOVA was used for unc104 OE data as all lines were in the same genetic background.


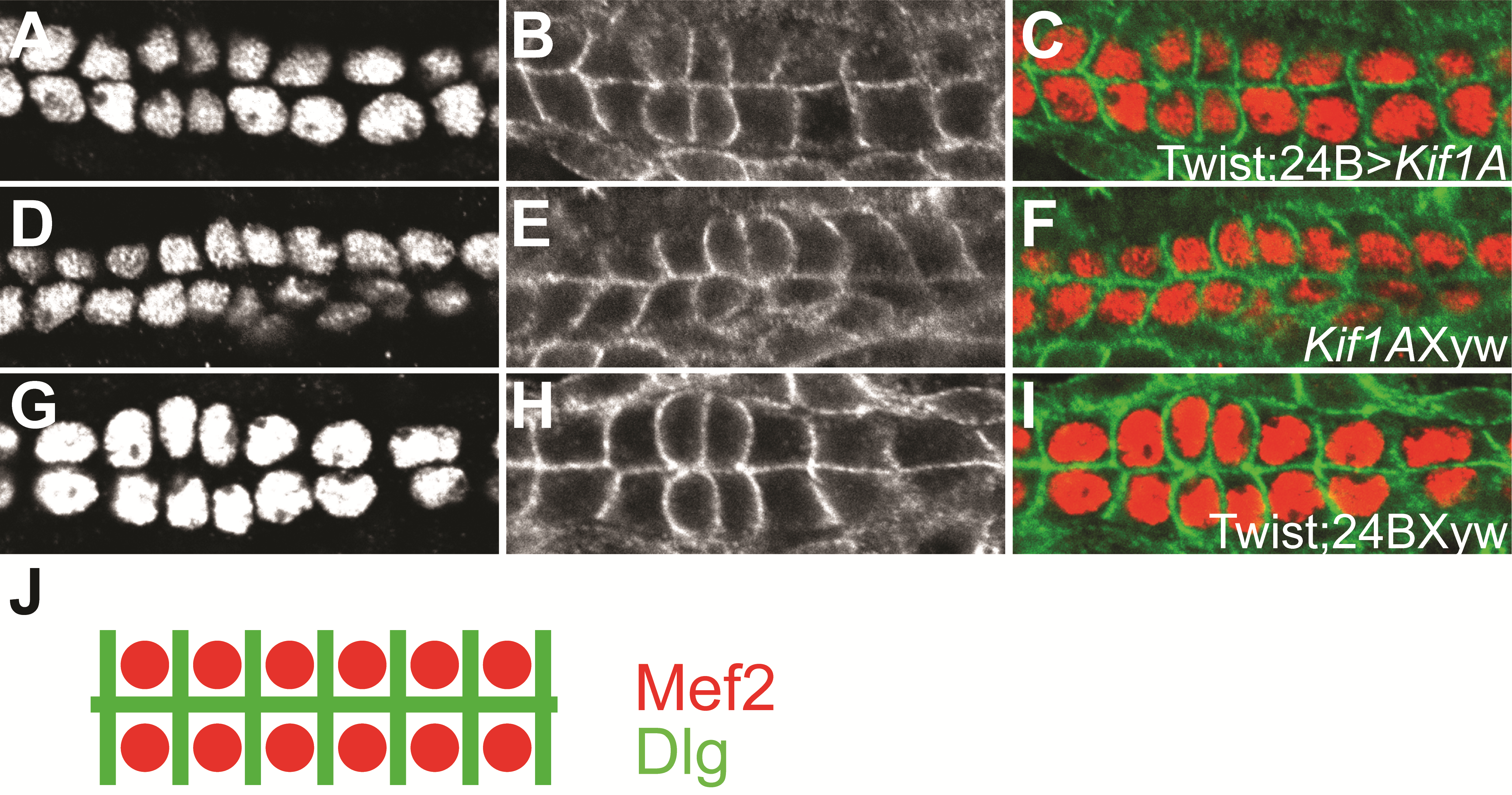


**Figure S4.** Cardiac primordial cell arrangement is not affected during embryogenesis. (**A**,**D**,**G**) Mef-2 staining (**B**,**E**,**H**) Dlg (disk large) staining, (**C**,**F**,**I**) merged images in stage 18 embryos. Note that the cell polarity marker Dlg in *KifAa* over-expression flies (**A**–**C**: Twist;24B > *Kif1A*) is localized to the dorsal-lateral sides of the heart. A similar distribution is observed in genetic controls (**D**–**F**: *Kif1A* X yw, and G-I: Twist;24B X yw). (**J**) Schematic diagram illustrating the normal distribution pattern of Dlg in stage 18 embryos.


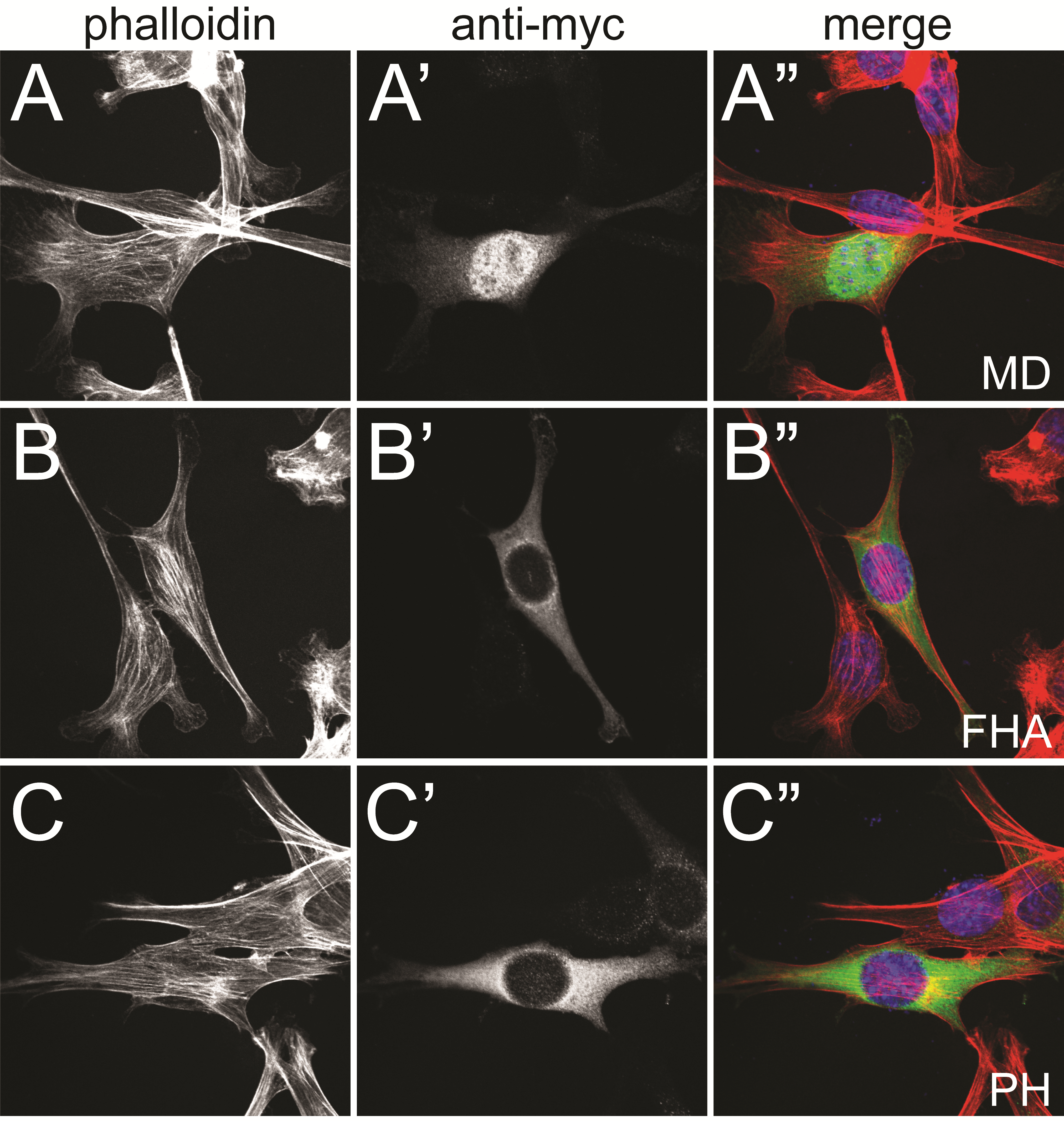


**Figure S5.** Actin cytoskeleton is not affected by over-expression of the individual *Kif1A* functional domains. (**A**) C2C12 cells transfected with MD with myc, (**B**) FHA tagged with myc, or (**C**) PH tagged with myc. The constructs used in this study are illustrated in Figure 6. The cells were stained with phalloidin (left panel **A**–**C**, red) and anti-myc antibody (middle panel **A**′–**C**′, green). Note that *Kif1A* MD is localized in the nucleus (**A**′).


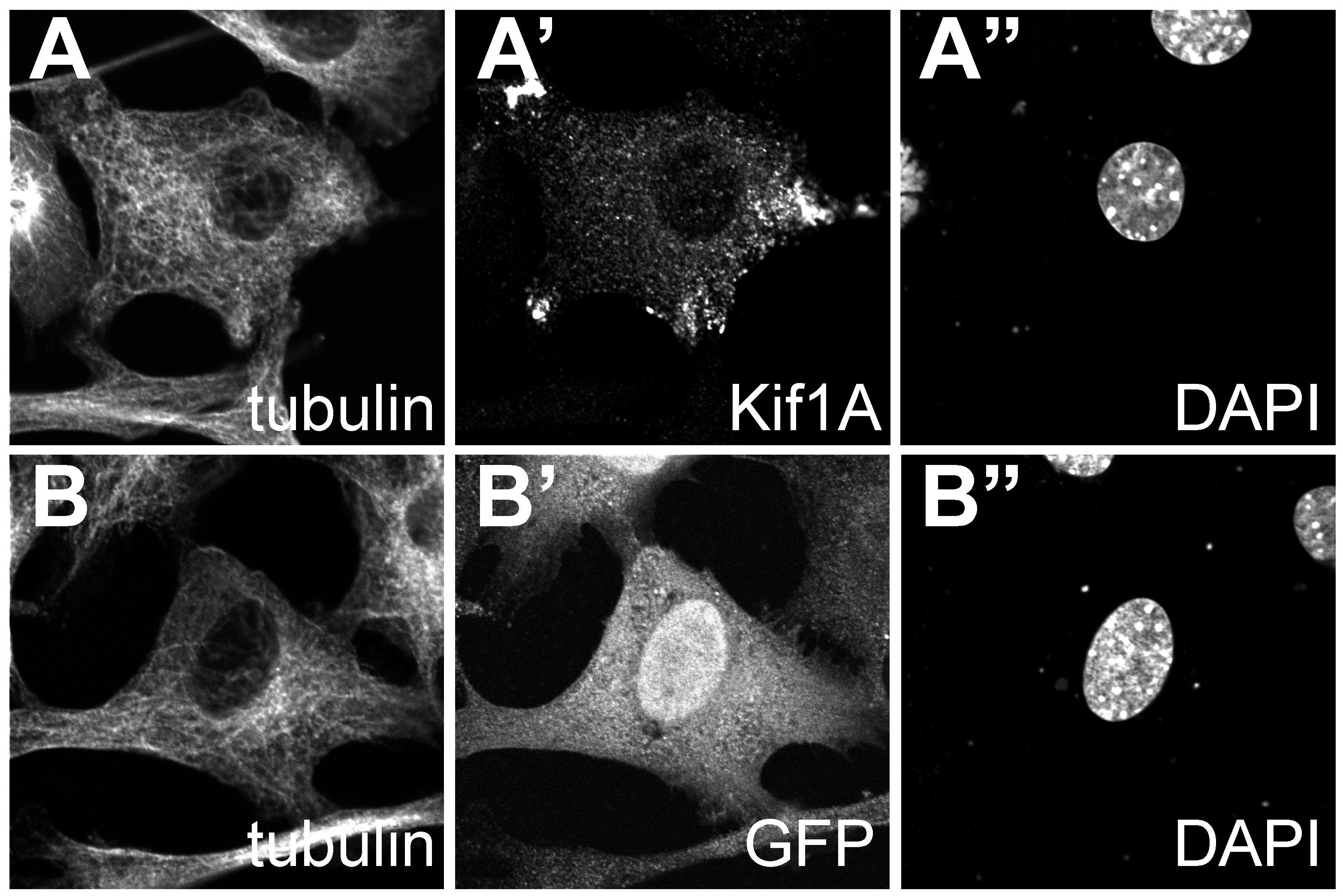


**Figure S6.** Microtubule structure is unaffected by *Kif1A* overexpression. (**A**) *Kif1A* over-expressing C2C12 cells showed no morphological changes in microtubules compared to (**B**) control cells overexpressing GFP. Microtubules stained with antitubulin (Abcam). These results suggest that *Kif1A* pathogenesis is primarily due to its effect on actin organization.


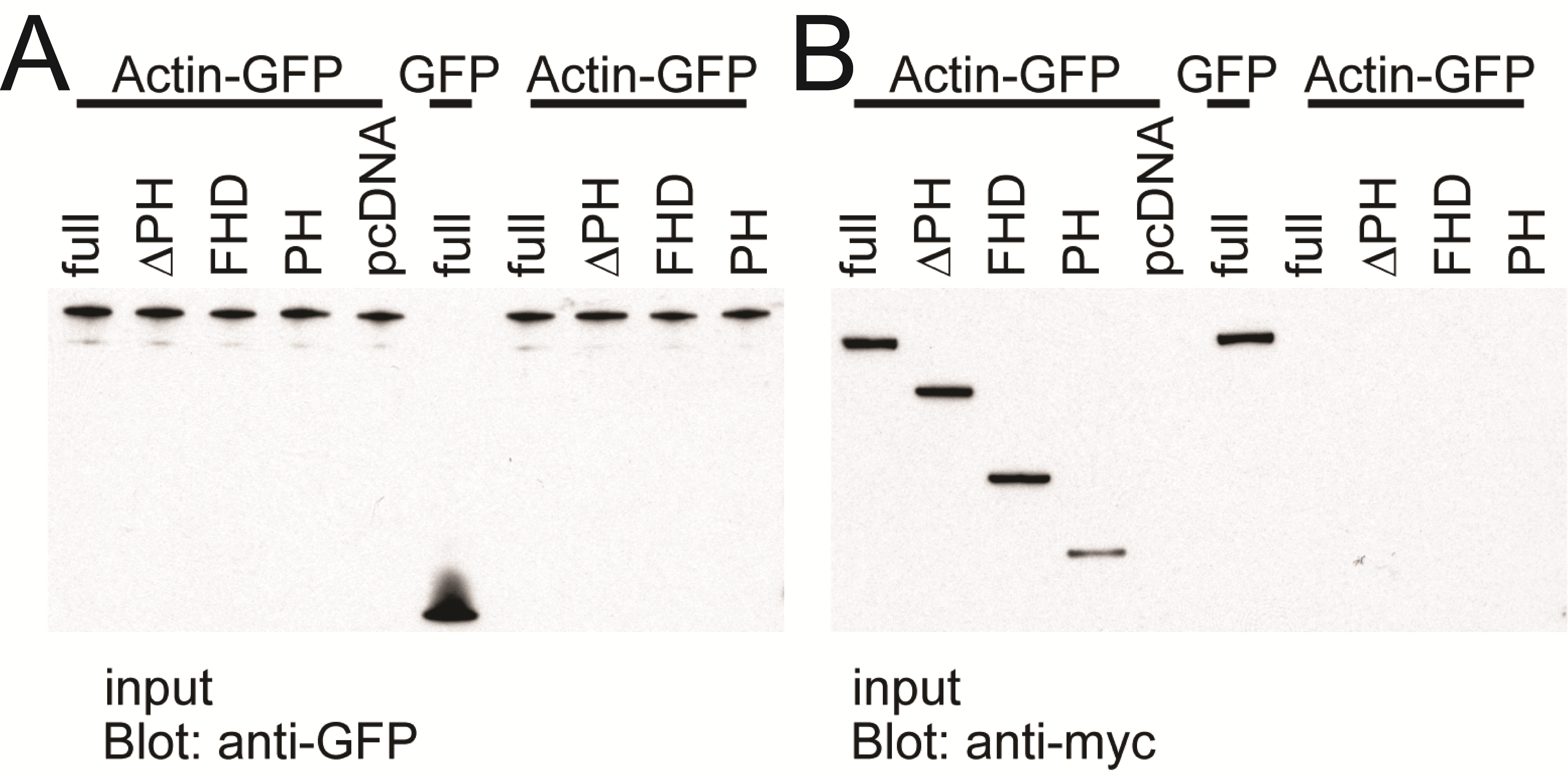


**Figure S7.** Kif1A physically associates with G-actin in myocytes. The inputs for text figure 5H. (**A**) Lysates from co-transfected HEK293 cells with Actin-GFP and full length or truncated *Kif1A* were detected by anti-GFP antibody to show that all samples were equally expressed actin-GFP. (**B**) The same lysates were detected by anti-myc antibody to show full length, Δ PH, FDH and PH proteins were all expressed at similar levels.


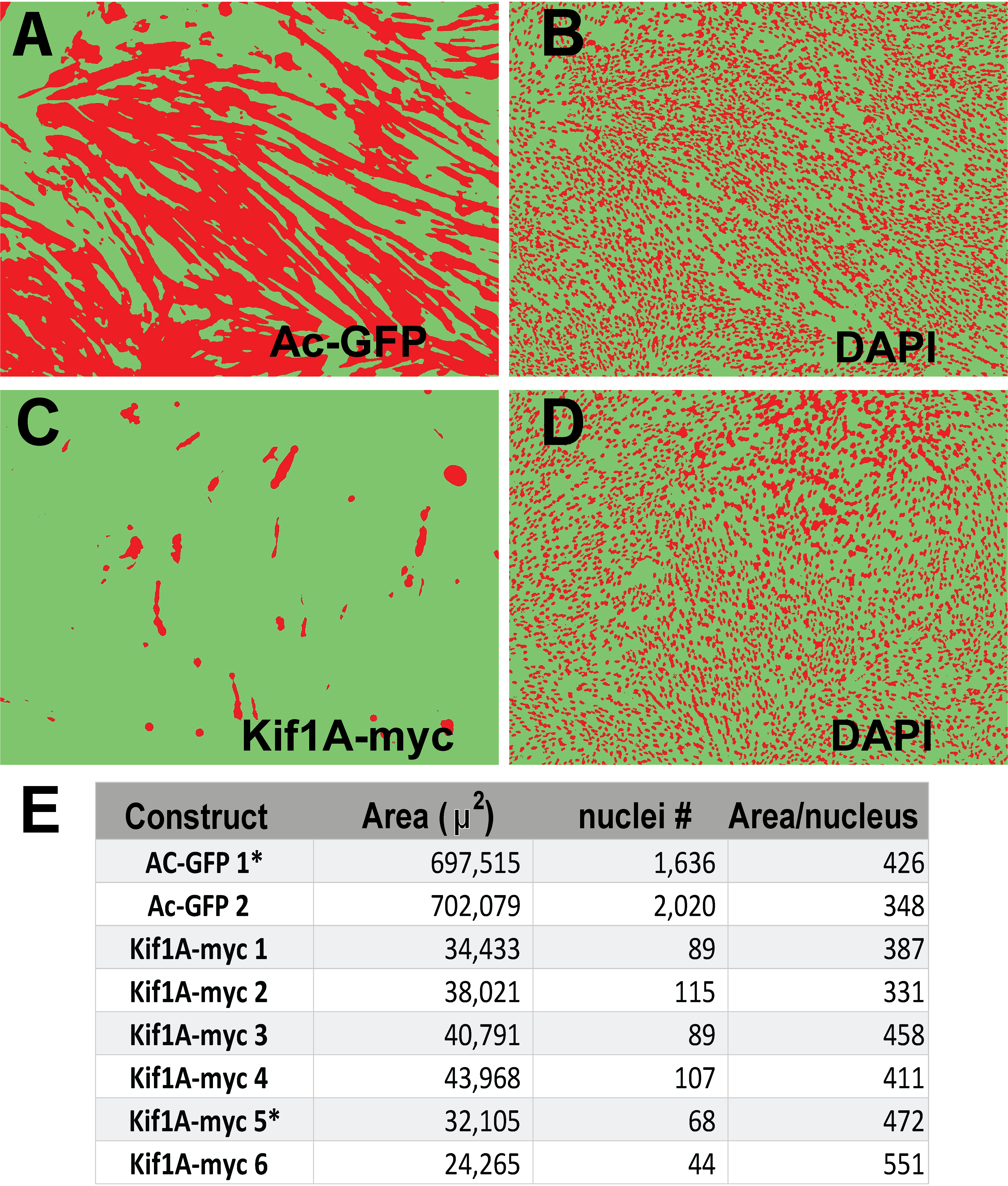


**Figure S8.** Quantification of *Kif1A* transfected C2C12 induced myocytes. Micrographs of stained induced myocytes (see Figure 7) were processed with the Trainable Weka Segmentation plugin in ImageJ to identify myocytes and nuclei (red). (**A**) Myocytes formed from Ac-GFP transfected C2C12 cells. (**B**) DAPI stained nuclei from the same dish as in (**A**). (**C**) Myocytes formed from *Kif1A*-myc transfected C2C12 cells. (**D**) DAPI stained nuclei from the same dish as in (**C**). (**E**) Summary table showing the total area of transfected cells and the number of nuclei associated with the transfected cells. *Images from these cell transfections are shown in **A**–**D**.

**Movie S1–High-speed movies of in situ Drosophila hearts.**

**Wt heart:** The top movie shows a heart from a wildtype fly that is contracting rhythmically. (The red dots identify the pixels used by the SOHA algorithms to quantify movement and also highlight the heart edges.) The locations of ostia (inflow tracts) and positions of the internal valve between chamber 2 & 3 are indicated in all movies. Note the absence of valve structures separating chambers 2 and 3 in the bottom 2 movies.

**Kif1A OE hearts:** The bottom three movies are from flies overexpressing Kif1A in all mesoderm including the heart myocardial cells (24B-gal4 > UAS-Kif1A). The second movie shows heart valve dysplasia with dysplastic-appearing valve structures that do not coapt normally. The third movie shows a more extreme example. In the bottom movie there are no visible valve structures and the heart does not contract at the location where those structures are normally found.
